# Effects and Correction of Patient Bulk Motion in Cranial DENSE MRI

**DOI:** 10.1002/mrm.70270

**Published:** 2026-02-01

**Authors:** Caroline A. Doctor, Leonardo A. Rivera-Rivera, Laura B. Eisenmenger, Sterling C. Johnson, Kevin M. Johnson

**Affiliations:** 1 Department of Medical Physics, University of Wisconsin, Madison, Wisconsin, USA; 2 Department of Medicine, University of Wisconsin, Madison, Wisconsin, USA; 3 Department of Radiology, University of Wisconsin, Madison, Wisconsin, USA; 4 Department of Biomedical Engineering, University of Wisconsin, Madison, Wisconsin, USA

**Keywords:** brain, brain tissue motion, DENSE, displacement imaging, motion correction

## Abstract

**Purpose:**

Applications of DENSE to measure cardiac driven brain tissue pulsations are highly sensitive to bulk patient motion due to the sub-millimeter displacement encoding required, limiting its accuracy, reproducibility, and use in pediatric and aging populations. This study aims to assess the impact of induced bulk motion on DENSE scans and to what extent these motion effects can be mitigated with existing and newly proposed methods.

**Methods:**

Participants (*N* = 10) underwent test–retest 2D DENSE scans at three slice locations with and without induced motion using a 3.0 T system. Brain displacement fields were calculated using pipelines without and with motion correction based on polynomial fitting to an outer ring of brain tissue. Subsequently, voxel-wise comparisons were made between scans and pipelines to evaluate scan repeatability and measure biases in displacement measures.

**Results:**

In comparing scans with and without induced motion, motion significantly impacted displacement measures, resulting in intensity variations and phase wrap artifacts, as well as increased the mean peak-to-peak displacements. Motion-correction removed the intensity variations and phase wrap observed in phase images, and reduced variations between scans taken with and without induced motion ($R_{\text{corr}.}^{2}=0.45\pm 0.29,\;R_{\text{corr}.}^{2}=0.96\pm 0.05$; RMSD_uncorr_. = 0.089 ± 0.005 mm, RMSD_corr._ = 0.00788 ± 0.00004 mm). Test–retest reproducibility increased after motion correction with induced motion (*ρ*_uncorr_. = 0.46 ± 0.38, *ρ*_corr._ = 0.98 ± 0.01), and in the absence of induced motion (*ρ*_uncorr_. = 0.76 ± 0.35, *ρ*_corr._ = 0.98 ± 0.02).

**Conclusion:**

Motion correction significantly improved the correspondence of DENSE measures acquired during induced motion and improved test–retest reproducibility, even in the absence of induced motion.

## 1 | Introduction

Aging populations exhibit biomechanical and morphological changes including decreased brain tissue volume [1–15], increased ventricular volume [2, 5, 9, 12, 13, 16], increased arterial stiffening [17–25], and a decrease in whole brain averaged stiffness [26–30]. The interaction of these changes is hypothesized to contribute to the tissue damage and pathology of disease such as dementia and hydrocephalus. For example, arterial stiffening increases the amplitude of the pressure wave being transmitted into the capillary beds and the resultant experienced strain in tissue is affected by local and global brain tissue stiffness.

Brain tissue displacements also propagate to drive interstitial fluid, and cerebral spinal fluid (CSF) flow through the ventricles, cerebral aqueduct, and spinal canal [31–34]. Cognitive decline has been linked to both decreased brain tissue viscoelasticity [35, 36] and increases in vascular stiffening [37–50]. Decreased brain tissue viscoelasticity has also been linked to decreased neuronal density [51], increased demyelination [52], and chronic neuroinflammation [53, 54]. Despite these observations, the mechanisms by which brain biomechanics affect brain function and disease remain poorly understood and there is a need to further characterize these properties in human populations, particularly effects in tissue.

MRI provides multiple methods to image brain tissue biomechanical properties and dynamics. Of particular interest are tissue properties such as stiffness, and the magnitude and direction of the intrinsic cardiac-driven motion, from which estimates of tissue strain can be derived. Magnetic resonance elastography (MRE) using external drivers provides means to measure tissue stiffness [26–28, 30, 35, 52, 53, 55–80] and is the current standard method for in vivo measures but cannot provide measures of intrinsic motion. MRI is capable of measuring the intrinsic motion of the brain using Displacement Encoding with Stimulated Echoes (DENSE) [81–90], phase contrast (PC-MRI) [31, 32, 91–94], and quantitative-amplified MRI (q-aMRI) [95–100]. DENSE is of particular interest due to its high level of sensitivity to small, cardiac driven motions in the brain and its ability to directly provide quantitative values [81]. While DENSE was originally developed for cardiac applications to measure myocardial motion on a millimeter-level scale [101–106], DENSE also provides methodology to non-invasively measure sub-millimeter, cardiac-induced brain tissue displacements [82–90]. DENSE measures tissue displacement maps over the cardiac cycle, which can be processed to measure brain strain and tissue stiffness using intrinsic magnetic resonance elastography.

Prior studies utilizing DENSE in the brain [82–90] have largely focused on technical development in younger, healthy participants (ages 19–42 years) and confounding effects found in clinical populations have not yet been fully investigated. Many target applications of DENSE are in aging and pediatric populations, and these populations are known to have high incidences of bulk head motion [107–111]. The cardiac driven displacement of the brain is sub-millimeter with most studies using a sensitivity of 0.5 mm/cycle [31, 84, 91, 92, 112]. Thus, implementations of DENSE in the brain are inherently sensitive to bulk brain motion, similar to phase effects observed in diffusion MRI. Larger amounts of bulk motion can lead to phase wrap that is difficult to identify and correct. For these reasons, most DENSE processing chains incorporate background phase corrections and/or unwrapping algorithms [82–90], yet the efficacy of these techniques remains under investigated.

This study aims to understand the effect of induced bulk motion on DENSE-based measures of brain displacement. Further, the study aims to compare existing and a newly proposed method for correcting bulk motion effects. This is based on the premise of removing confounding effects to brain displacement measures to enable expanded studies in aging and pediatric populations.

## 2 | Methods

To evaluate the impact of induced bulk motion on DENSE scans in the brain, a test–retest imaging study was performed with and without subtly induced bulk motion. Resulting DENSE images were post-processed using pipelines without and with additional motion correction methods, and displacement values were compared across the acquisitions and correction methods. The effectiveness of motion correction was evaluated based on differences in test–retest agreement between scans with and without induced bulk motion. Code is available at https://github.com/c-doctor/DENSE_2DMotionCorrection.

### 2.1 | Imaging Protocol

A prospective study was performed on 10 healthy participants (3 female; ages 23–41) with the overall goal of characterizing motion effects and evaluating a motion correction method. While this cohort may not represent clinical populations, it was selected based on a higher likelihood of achieving baseline scans without incidental bulk head motion. All study activities were performed after obtaining informed written consent and approved by the local institutional review board. Each participant was scanned using a 3.0 T MR scanner (Signa Premier, GE Healthcare), equipped with a 48-channel head coil and photo-plethysmography gating (PPG). Additional padding was used to restrict excess head motion. Participants underwent a single MRI visit with a series of 2D DENSE scans collected.

Localizer images were used to prescribe 2D axial slices at three different slice locations: mid-ventricles, top edge of ventricles, and ^~^1 cm superior to the ventricles. Slice positioning was chosen based on anatomical landmarks within each individual and remained fixed across the scan session for each individual. DENSE datasets acquired were 2D, single-shot, single-slice, with a spiral readout. In-plane resolution was 4 mm × 4 mm, and a slice thickness of 10 mm was chosen to ensure sufficient SNR. The in-plane FOV was 24 cm × 24 cm, bandwidth of ±125 kHz, 4.5 ms echo time, and variable flip angle sampling [113, 114]. A balanced four-point displacement encoding strategy [115] was used and phase cycling was utilized to yield eight directions of encoded motion. For each scan, four averages were performed. The time between echo images after cardiac triggering was calculated using PPG heartrate data (at time of prescription) so that 10 stimulated echoes covered 110% of the cardiac cycle (73.34 to 132.00 ms temporal resolution for heartrates between 90 and 50 bpm). Images were collected every 2 RR intervals. A total of 320 images were acquired in each acquisition: 10 images per preparation, 8 encoding directions, and 4 averages. This resulted in a scan-duration of 42.67 to 76.80 s depending on the heartrate (90 to 50 bpm respectively). A displacement encoding frequency of 2.02 cycles/mm was used in each of the three directions, giving a 0.495 mm/cycle displacement sensitivity.

Test–retest scans were performed at each slice location to assess repeatability, and scans were taken both with and without induced motion. Test–retest measurements used for the evaluation of reproducibility were performed back-to-back, and participants were not repositioned between any of the acquisitions, all using identical scan prescriptions. To induce bulk motion, participants were instructed to continuously move their feet in tandem (alternating plantar flexion and dorsiflexion), generating subtle bulk motion artifacts in those images. During scans without induced motion (baseline), participants were instructed to remain as still as possible.

### 2.2 | Post-Processing/Motion Correction

**FIGURE 1 |:**
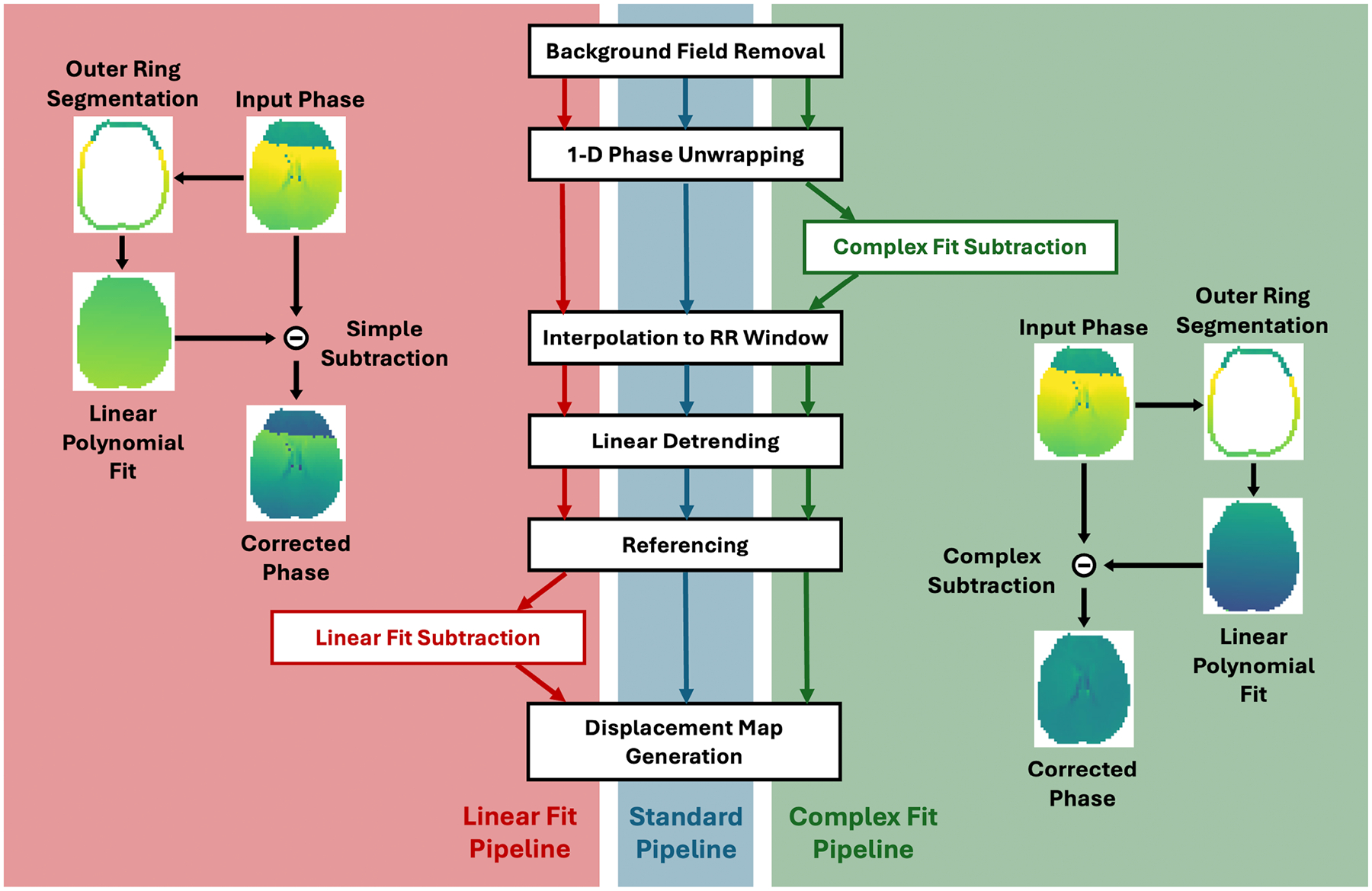
Visualization of the three post-processing pipelines used in the study. For clarification, the ‘Input Phase’ referenced in the visual representations of the Linear Fit Subtraction and Complex Fit Subtraction steps is the phase output by the previous step in the pipeline (indicated by their respective colored arrows in center of figure) and the ‘Corrected Phase’ is the phase passed on to the next step in the pipeline.

Reconstruction of the raw complex images was performed offline, using a non-uniform Fourier transform (NUFFT) and coil sensitivities estimated from low resolution images. All DENSE images underwent a standard post-processing pipeline consisting of background phase removal, phase unwrapping, linear detrending and referencing, prior to displacement map generation. Two alternate post-processing pipelines included all the steps of the standard pipeline as well as additional steps meant to aid in motion correction (Figure 1).

### 2.3 | Standard Pipeline

Background phase removal was performed on the complex images by multiplying all the images in a cardiac cycle by the complex conjugate of the first image in the cardiac series. Phase information was then extracted from the complex images. A simple 1D phase unwrapping along the time axis was performed, based on it outperforming a 3D variant of a 4D single-step Laplacian algorithm [116, 117]. The 10 phase images were linearly interpolated to 19 phase images over the cardiac cycle. PPG data collected during the scan was used to estimate the average RR interval during the acquisition. This estimated average RR interval was used to retrospectively correct for any mismatches in the heartrate at time of prescription from the scan by removing excess images from the end of each cardiac cycle. Voxel-wise linear detrending was performed over the time axis, to aid in removing some bulk motion, assuming the motion maintained a constant velocity throughout the entire cardiac cycle. All images in a cardiac cycle were then referenced to the first image of the series, so they all start and end with net zero displacement. All phase series were then multiplied by an encoding matrix to generate displacement maps.

### 2.4 | Motion Correction Method

DENSE encodes displacement in the phase of the MR image, resulting in no phase accumulation if the tissue remains stationary during the mixing period between magnetization tagging and readout. To enable motion correction, this study assumes rigid body head motion and that the outermost ring of brain tissue remains relatively stationary. The mechanical constraint imposed by the skull restricts displacement of voxels at the brain’s periphery. While capillary bed expansion may cause local deformations within edge voxels, such deformations would propagate inward, displacing more central voxels and leading to greater overall displacement in the brain’s interior compared with the periphery—supported by prior work [83–86, 115]. Consequently, voxels near the edge would exhibit minimal phase accumulation, so any phase detected in this region may be attributed primarily to bulk head motion. Assuming rigid body motion, a translation will result in a global phase offset based on the direction of the translation and the direction of the displacement encoding for the given shot. Rigid body rotational displacement similarly will result in a 1st order spatial offset in position based on the rotation matrix (e.g., *x*′ = *Ax* + *By* + *Cz* + *D*, where *A* − *D* describe the rotation). In 2D DENSE, this will result in a similar linear offset over the object being imaged with parameters determined based on the axis and degree of rotation and direction and magnitude of encoding. Examples of phase offsets for typical motion amounts are included in provided code. Therefore, a combination of translational and rotational motion should result in phase offsets that can be adequately fit using a first-order polynomial. For this study, the left and right hemispheres were segmented using a magnitude image, before being combined into a single mask. The outermost ring of voxels in the combined hemisphere mask was used to create a single voxel wide mask of the outer ring of brain tissue. For each phase image, a first-order polynomial fit of the phase in the segmented outer ring region was performed, before subtracting the resultant fit from the overall phase image, essentially removing the phase accumulation due to bulk motion from the image. This fitting was performed on each individual image, allowing for the correction of motion throughout the cardiac cycle.

### 2.5 | ‘Linear Fit’ and ‘Complex Fit’ Pipelines

Two post-processing pipelines were included for comparison to the Standard Pipeline. A ‘Linear Fit’ pipeline, which did not correct for residual phase wraps, and a ‘Complex Fit’ pipeline, which was designed to do so. In the Linear Fit pipeline, the phase within the outer ring was fit using an Ordinary Least Squares (OLS) linear regression model (scikit-learn, v1.7.2). The fit was applied at the end of the processing chain, following all other post-processing steps and prior to displacement map generation. The phase map derived from the optimal linear fit was subtracted from the overall phase image to remove residual bulk motion before displacement maps were generated. The Complex Fit pipeline employed a two-stage optimization procedure. An initial coarse grid search was used to identify slope and intercept values minimizing the error between the phase in the outer ring and a first-order polynomial model adjusted for phase wrapping. These parameters served as initial estimates for local refinement using the scipy.optimize.minimize function (SciPy, v1.16.0). The resulting best-fit phase map and the phase image were then represented in the complex domain by mapping each voxel’s phase to a complex number on the unit circle via Euler’s formula. Subtraction of the fitted phase from the phase image was performed in the complex domain, removing bulk motion contributions. The corrected phase was obtained by extracting the angular component of the resulting complex number. This correction was performed following the unwrapping step in the Standard Pipeline and prior to interpolation of additional images across the cardiac cycle, as interpolation could alter phase differences in regions with residual phase wrap, rendering complex subtraction ineffective.

### 2.6 | Displacement Map Generation

Displacement maps for motion in the Anterior/Posterior (A/P), Left/Right (L/R), and Superior/Inferior (S/I) directions were output after multiplication of the corrected phase images with the displacement encoding matrix. Magnitude images were used to draw ROIs of the left and right hemispheres. For voxels within the left and right hemispheres, the magnitude of the 3D displacement vector over the cardiac cycle was calculated using the generated A/P, L/R, and S/I displacement maps. Additionally, the maximum difference in the 3D displacement vector between all sampled timepoints over the cardiac cycle (i.e., the range of cardiac resolved displacement), referred to as the peak-to-peak displacement in this work, and was found for voxels within the left and right hemispheres.

### 2.7 | Statistics and Comparisons

For each participant, slice, and pipeline, a mean peak-to-peak displacement was found by averaging the peak-to-peak displacement measures across voxels within the left and right hemispheres. A maximum peak-to-peak displacement measure was found by averaging over the highest 5% of peak-to-peak displacement measures within the left and right hemispheres. Averaging between test–retest scans and treating each participant and slice position independently, displacements in the A/P, L/R, and S/I directions averaged across the left and right hemispheres were plotted with respect to the cardiac cycle, to show the shape of the displacement curve over the course of the cardiac cycle for each post-processing pipeline. Data was then separated into pairs, containing one scan acquired with induced motion and an identical scan acquired without induced motion (baseline). Linear regressions were performed for each pair using the voxel-wise magnitude of the 3D displacement vector maps, and the Root Mean Square Deviation (RMSD) was calculated for each pair as well. To assess repeatability, Spearman’s Rank Correlation coefficients were calculated for test–retest scans with and without induced motion, with each participant and slice position treated independently. As an additional assessment of repeatability, linear regressions were performed between test–retest scans, treating scans with or without induced motion, participants, and slice positions independently. The Wilcoxon signed-rank test was performed on the mean and maximum peak-to-peak displacement measures within the hemispheres to determine their statistical significance before and after additional motion correction methods. Significance was set to be 0.05. Since three slices were taken for each participant, Bonferroni corrections were applied to the *p*-values after calculation. The heartrate variability of each participant during each acquisition was assessed using the Root Mean Square of Successive Differences (RMSSD) based on raw PPG data collected during the scan.

Secondary analysis of the peripheral motion in baseline and induced motion scans was performed to validate the assumption that the outermost ring of brain tissue remains stationary, included in the Supporting Information. Erosion of the hemisphere masks was used to produce masks of the interior brain regions. Peak-to-peak displacement magnitudes were compared before and after application of motion correction methods, between interior and peripheral regions. Reconstructing the four acquired averages separately, displacements within the periphery were plotted with respect to the cardiac cycle, allowing for comparison between repeated measures.

## 3 | Results

### 3.1 | Motion Effects on DENSE Without Motion Correction

**FIGURE 2 |:**
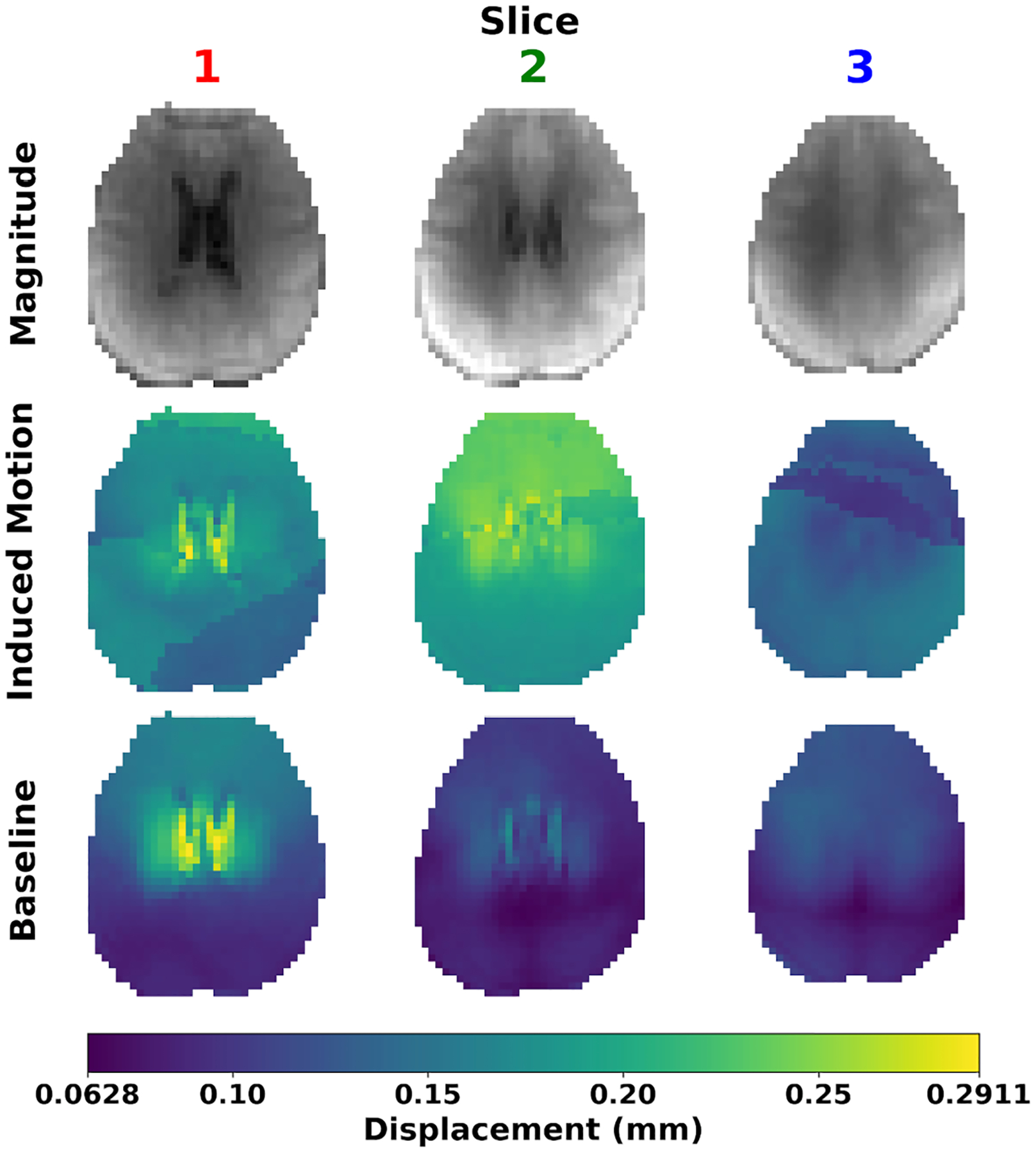
Magnitude images and peak-to-peak displacement maps at all three imaged slice locations, demonstrating the impact uncorrected bulk motion can have on the resultant displacement maps.

Figure 2 shows exemplary DENSE images at the three slice locations with and without induced motion. Images taken during induced motion showed quantitative and qualitative differences when compared with those without induced motion (baseline). The mean peak-to-peak displacement over both hemispheres increased from 0.09 ± 0.03 mm (baseline) to 0.17 ± 0.07 mm (induced motion). Scans with induced motion often showed residual phase wrap artifacts in the generated displacement maps, not present in the absence of induced motion.

### 3.2 | Effects of Motion Correction on DENSE

**FIGURE 3 |:**
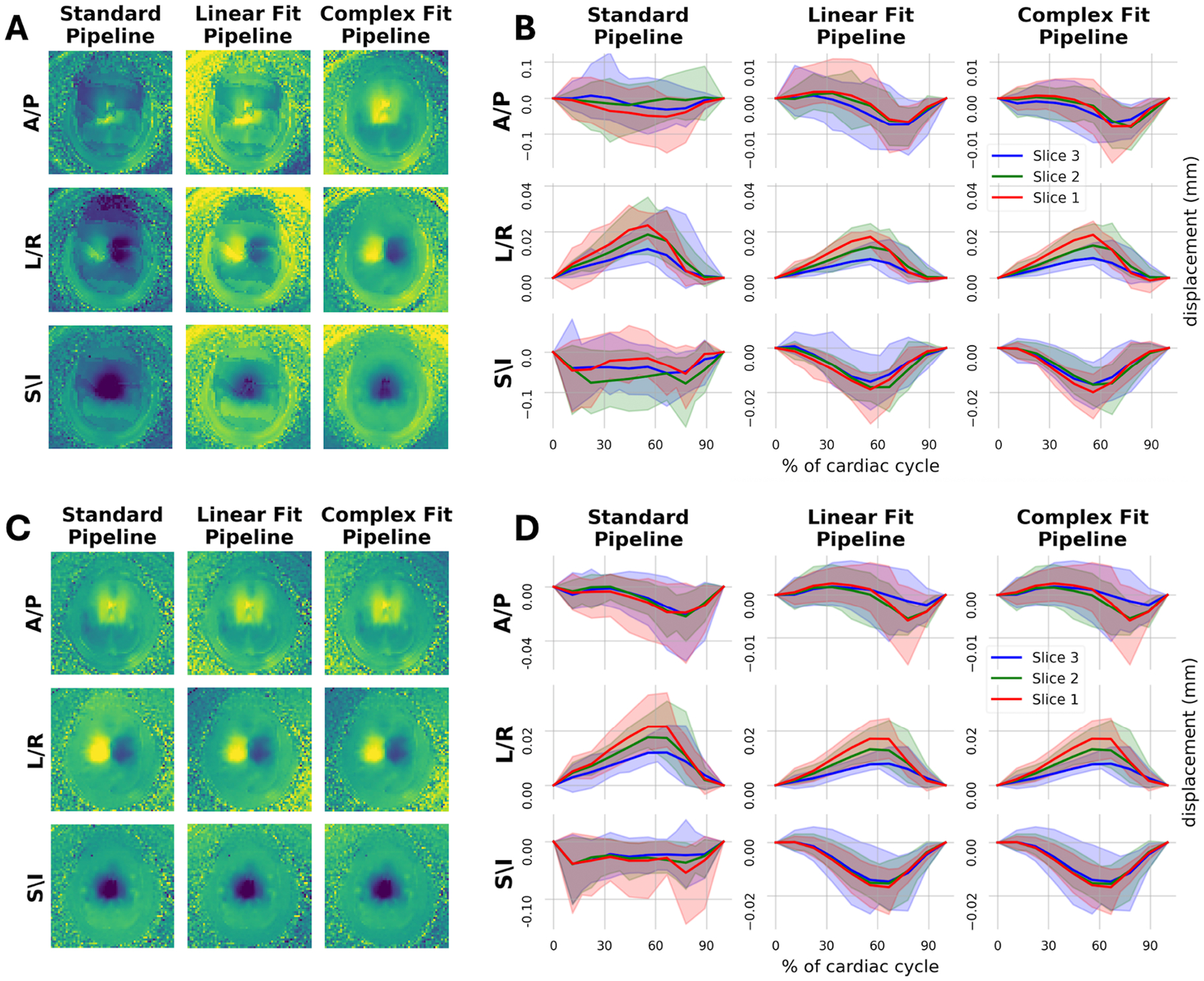
Effects of motion correction from a qualitative standpoint, with visual impact on displacement maps in parts (A and C), and visual impact on displacement curves in parts (B and D). (A) Example case with induced motion showing resultant displacement maps of all three orthogonal directions after each pipeline. (B) Displacement curves over the cardiac cycle of all three orthogonal directions after each pipeline for cases with induced motion. Note the *Y*-axis range is not consistent between the Standard Pipeline and the Linear Fit & Complex Fit Pipelines in the A/P and S/I directions. (C) Same example case as in part A, but without induced motion (baseline), showing resultant displacement maps of all three orthogonal directions after each pipeline. (D) Displacement curves over the cardiac cycle of all three orthogonal directions after each pipeline for cases without induced motion.

Figure 3 shows representative images of induced motion scans without and with the application of each of the motion correction pipelines. Both motion correction pipelines visually improved the correspondence between induced motion and baseline scans. Residual phase wrap artifacts were observed in the displacement maps for the Linear Fit Pipeline, but not the Complex Fit Pipeline (Figure 3A). Induced motion artifacts were primarily in the A/P and S/I directions, as expected by continuous alternating plantar flexion and dorsiflexion of the feet in tandem. The displacements in the A/P, L/R, and S/I directions, for scans with induced motion were averaged across each hemisphere and plotted with respect to the cardiac cycle (Figure 3B). The L/R curves are relatively similar for all three pipelines, in line with expectations. Both the A/P and S/I displacement curves improved substantially between the Standard and the Linear Fit Pipelines, with more subtle improvements observed between the Linear Fit and Complex Fit Pipelines. All further in-text analysis will focus on the improvements between the Standard and Complex Fit Pipelines. Further results for the Linear Fit Pipeline can be found in the Supporting Information.

**FIGURE 4 |:**
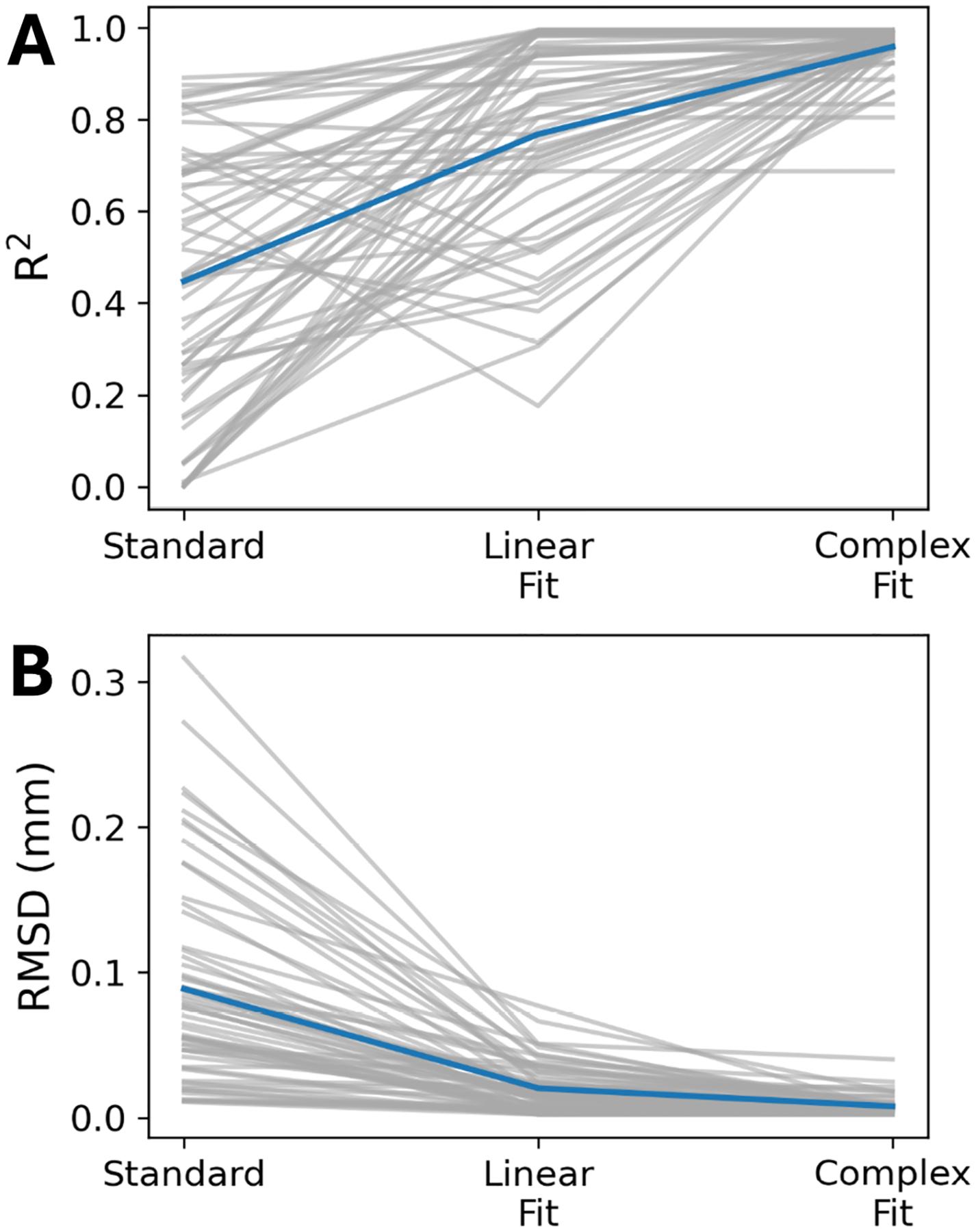
(A) Changes in *R*^2^ across the three post-processing pipelines. (B) Changes in the RMSD across the three post-processing pipelines. Gray lines are individual slice positions and participants, and the blue line is the average across all slice positions and participants.

**FIGURE 5 |:**
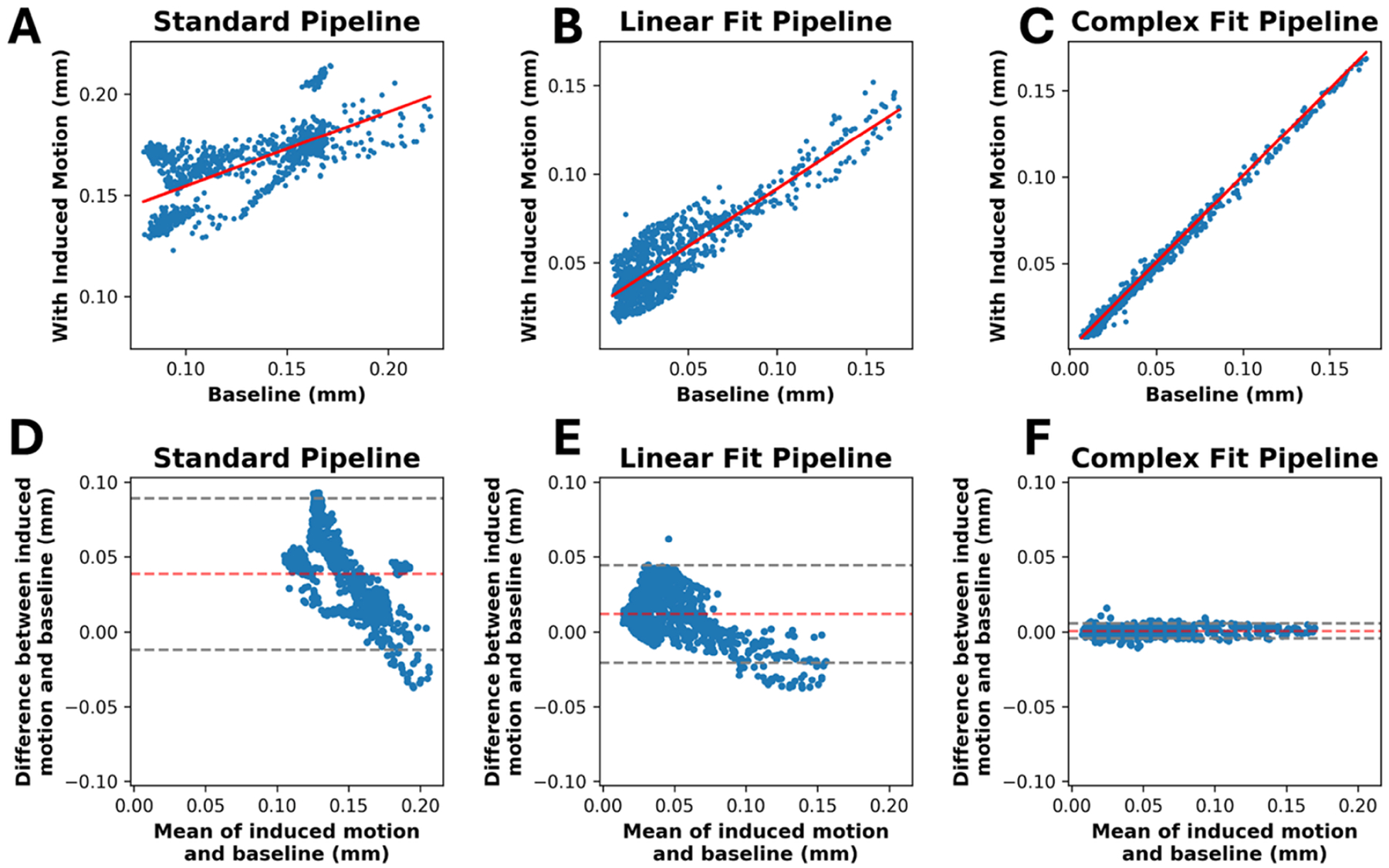
Linear regressions (A–C) and Bland Altman plots (D–F) for a representative case processed through each pipeline.

Data run through the Complex Fit Pipeline, when compared with data run through the Standard Pipeline, showed a 113.33% increase in the *R*^2^ value in linear regressions of induced motion and baseline pairs, from 0.45 ± 0.29 to 0.96 ± 0.05 (Figure 4A). After motion correction using the Complex Fit Pipeline, *R*^2^ values between induced motion and baseline scans remained relatively stable with increasing heartrate variability (assessed using the RMSSD), except for one participant, who exhibited significantly higher RMSSD and correspondingly lower average *R*^2^ values compared with the other participants (Figure S3B). We observed a 167.46% decrease in the RMSD between induced motion and baseline pairs, between the Standard and Complex Fit Pipeline (from 0.089 ± 0.005 mm to 0.00788 ± 0.00004 mm) (Figure 4B). A representative case showing the voxel-wise improvements is shown in Figure 5, demonstrating how each post-processing pipeline affected the linear regressions and Bland–Altman plots.

### 3.3 | Quantitative (Test–Retest Metrics)

**FIGURE 6 |:**
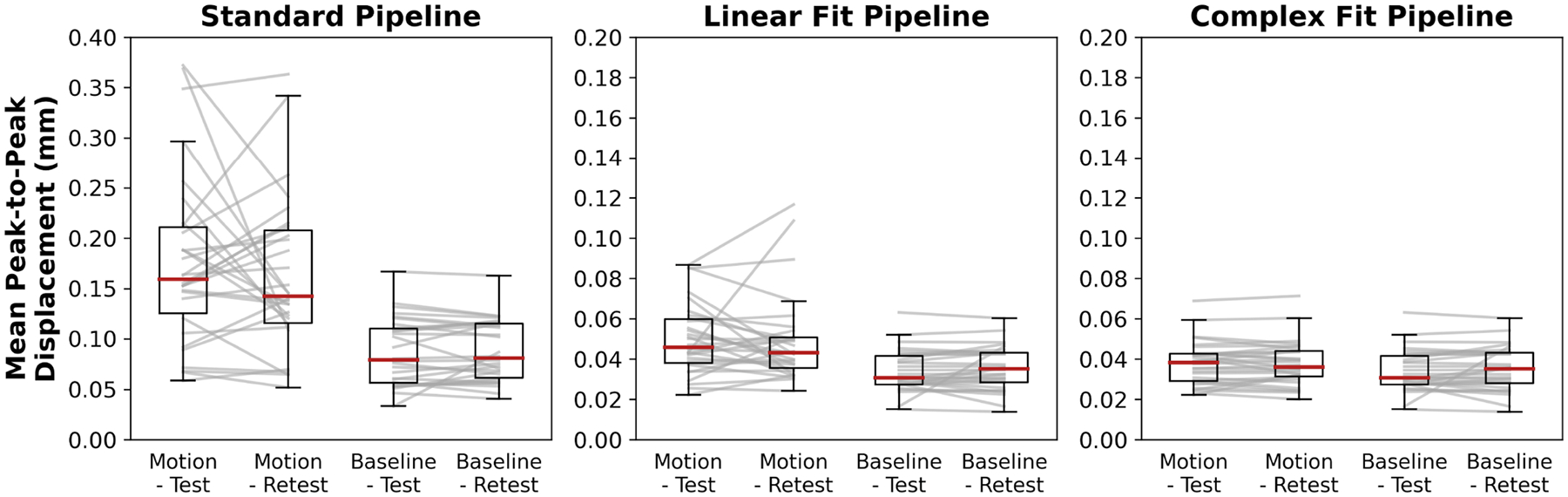
Boxplots of the mean peak-to-peak displacements after each pipeline across all participants and slice positions. Gray lines represent individual participants at individual slice positions to show variations in mean peak-to-peak displacement measures between test–retest scans. Y-axis range for the Standard Pipeline is double the range for the Linear Fit and Complex Fit Pipelines.

**FIGURE 7 |:**
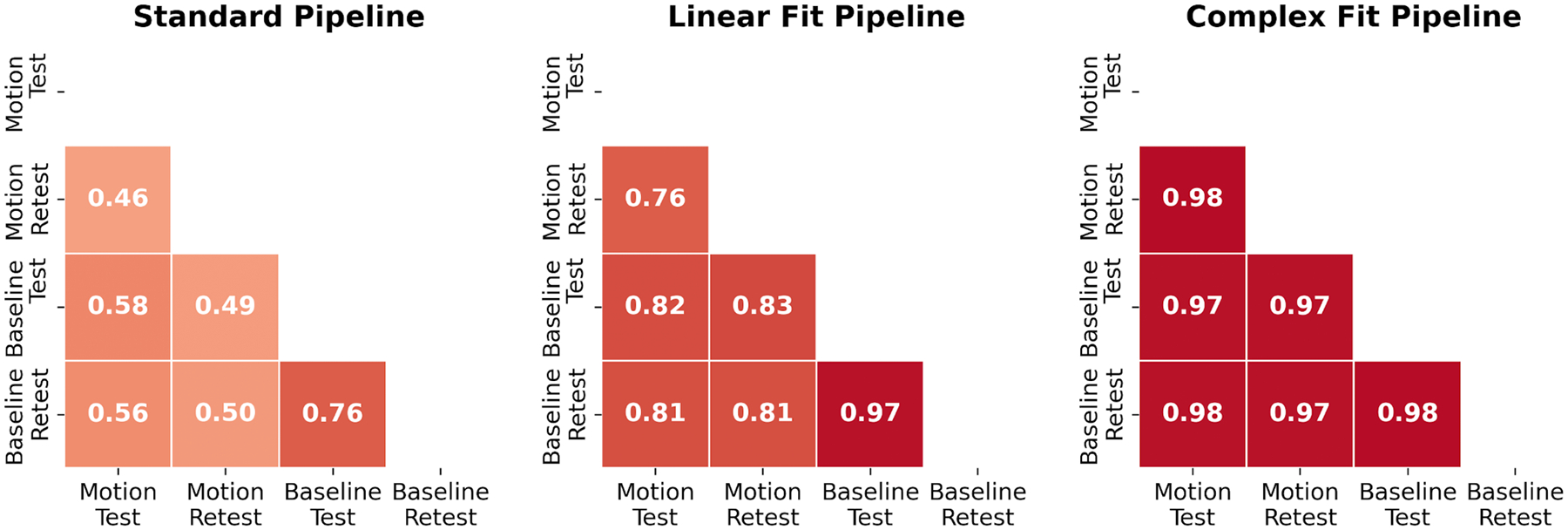
Heatmaps of Spearman’s Rank Correlation Coefficients demonstrating repeatability between test–retest, scans with induced motion and baseline scans for each pipeline. Averaged across participants and slice positions.

**FIGURE 8 |:**
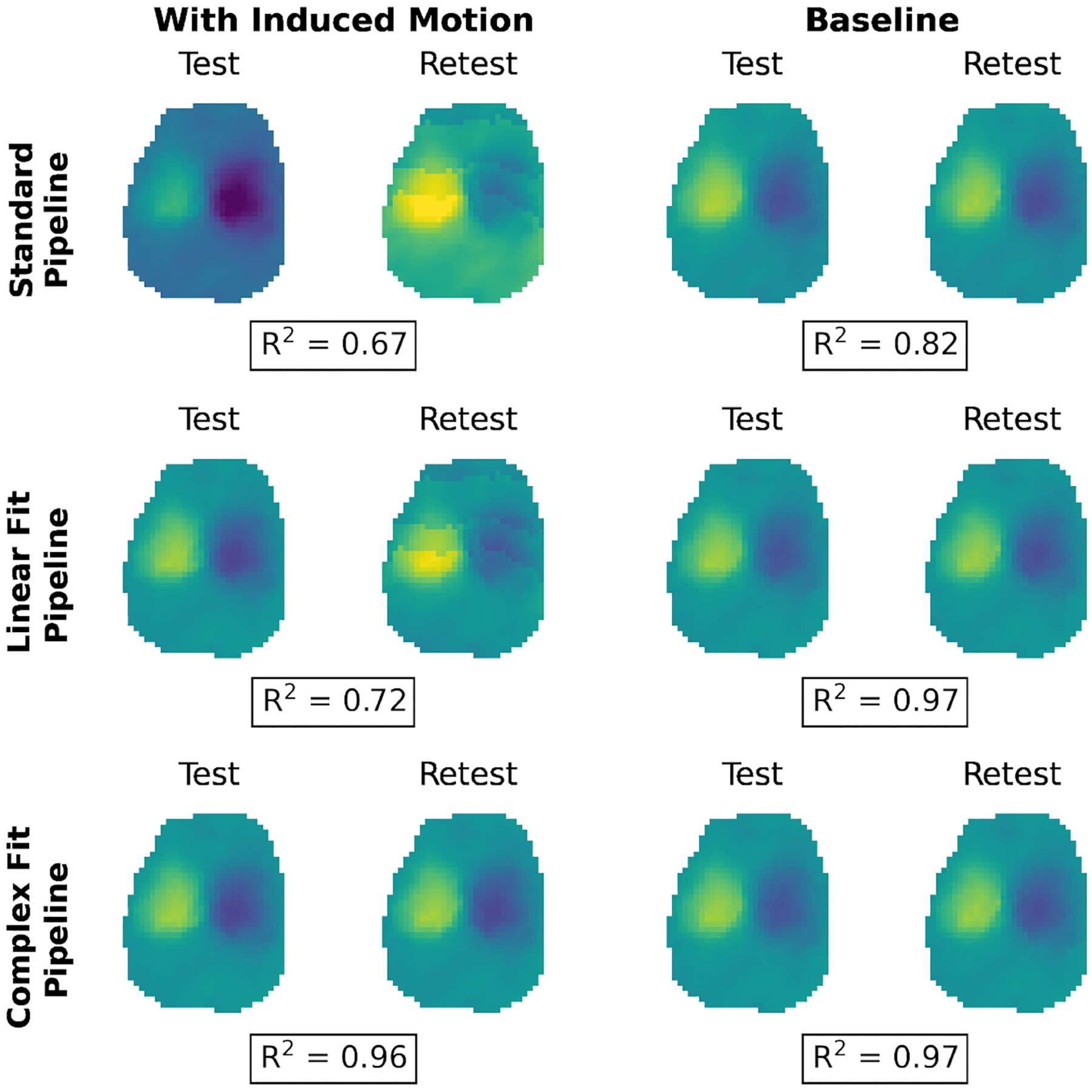
Visualization of L/R displacement maps in an example case showing variations between test–retest scans, for both scans with induced motion and baseline after each pipeline. The *R*^2^ value from a linear regression between test–retest scans is displayed underneath each pair.

Variations in peak-to-peak displacement measures between individual test–retest datasets decreased when motion correction methods were applied, with the greatest decrease in variability observed with the Complex Fit Pipeline (Figure 6). Spearman’s Rank Correlation coefficients between test–retest datasets averaged over all 10 participants and all three slice positions were 0.56 ± 0.01 with the Standard Pipeline and 0.977 ± 0.004 with the Complex Fit Pipeline. Delineating between test–retest scans acquired with and without induced motion, the Spearman’s Rank Correlation Coefficients for the data taken with induced motion were 0.46 ± 0.38 with the Standard Pipeline and 0.98 ± 0.01 with the Complex Fit Pipeline. The Spearman’s Rank Correlation Coefficients for the data taken without induced motion were 0.76 ± 0.35 with the Standard Pipeline and 0.98 ± 0.02 with the Complex Fit Pipeline (Figure 7). This pattern is also reflected in the linear regressions between test–retest datasets, with the greatest increase in *R*^2^ values being seen within the data acquired with induced motion, and smaller increases seen within the data acquired without induced motion (baseline), both resulting in *R*^2^ values close to 1.00, as demonstrated in an example case shown in Figure 8.

### 3.4 | End Displacement Results

**FIGURE 9 |:**
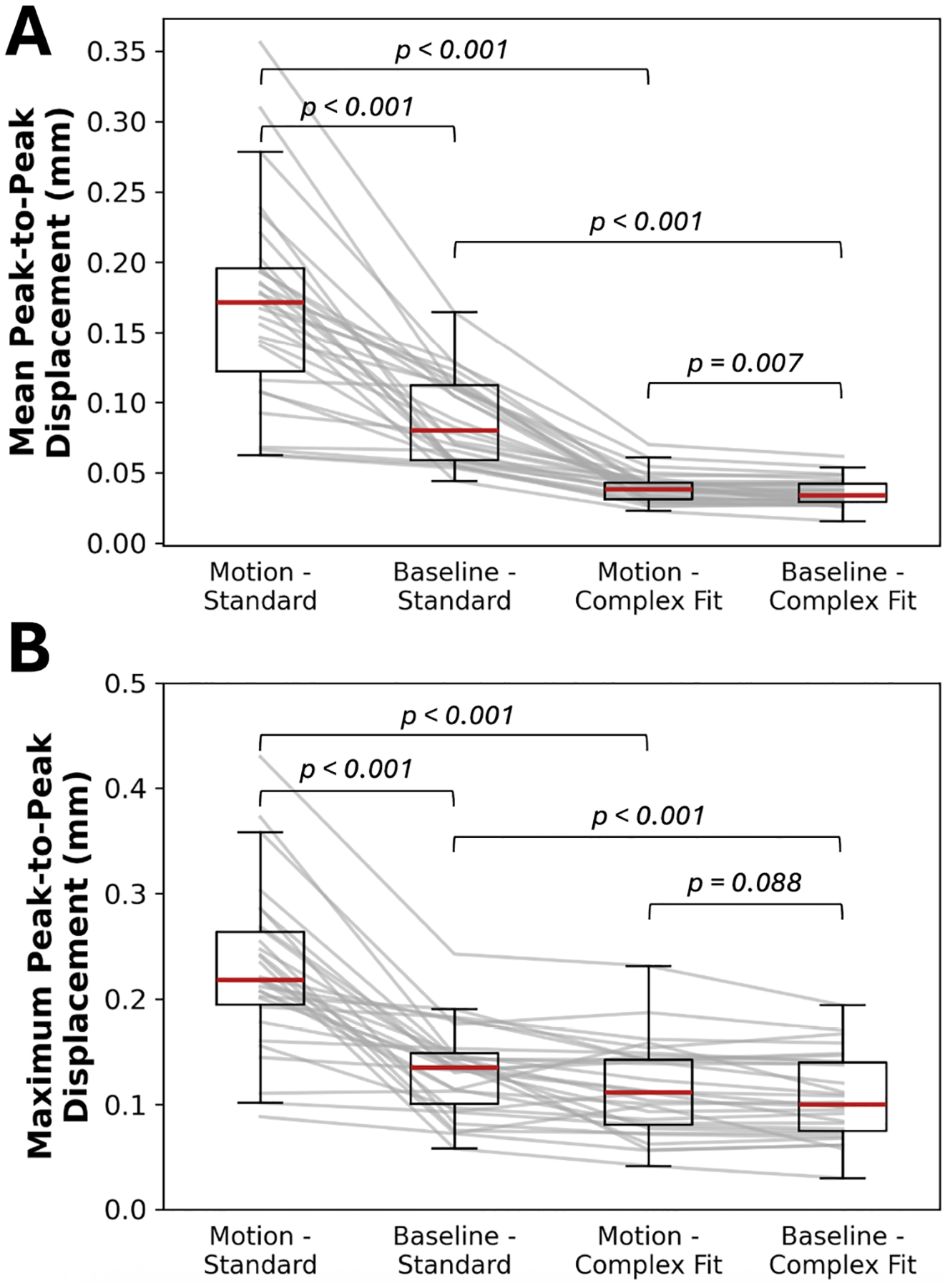
Changes in mean (A) and maximum (B) peak-to-peak displacement measures of scans with and without induced motion across all participants and slice locations, before and after motion correction methods. Annotations indicate the results of Wilcoxon signed-rank tests conducted on each paired combination, assessing the presence of statistically significant differences.

Mean peak-to-peak displacements across the hemispheres without any additional motion correction methods were 0.172 (IQR: 0.122–0.196) mm for acquisitions with induced motion, and 0.080 (IQR: 0.058–0.112) mm for acquisitions without induced motion. Maximum peak-to-peak displacements within the hemispheres without any additional motion correction methods were 0.218 (IQR: 0.195–0.263) mm for acquisitions with induced motion, and 0.135 (IQR: 0.101–0.149) mm for acquisitions without induced motion. After motion correction methods were applied (Complex Fit Pipeline), peak-to-peak displacements across the hemispheres were 0.038 (IQR: 0.031–0.043) mm (mean) and 0.112 (IQR: 0.080–0.142) mm (maximum) for acquisitions with induced motion, and 0.034 (IQR: 0.029–0.042) mm (mean) and 0.100 (IQR: 0.075–0.140) mm (maximum) for acquisitions without induced motion (Figure 9).

Mean peak-to-peak displacements for uncorrected acquisitions with and without induced motion were found to have statistically significant differences (*p*-value < 0.001). Mean peak-to-peak displacements for corrected acquisitions with and without induced motion were found to have statistically significant differences (*p*-value = 0.007). The effect size (*r* = 0.5389) indicates a moderately consistent residual difference, despite substantial overlap in the distributions. While statistically detectable, the absolute magnitude of this difference was small (median difference = 0.004 mm), and is unlikely to be clinically or biologically significant. Mean peak-to-peak displacements for uncorrected and corrected acquisitions with induced motion were found to have statistically significant differences (*p*-value < 0.001). Mean peak-to-peak displacements for uncorrected and corrected acquisitions without induced motion were found to have statistically significant differences (*p*-value < 0.001). Maximum peak-to-peak displacements for uncorrected acquisitions with and without induced motion were found to have statistically significant differences (*p*-value < 0.001). Maximum peak-to-peak displacements for corrected acquisitions with and without induced motion were found to not have statistically significant differences (*p*-value = 0.088). Maximum peak-to-peak displacements for uncorrected and corrected acquisitions with induced motion were found to have statistically significant differences (*p*-value < 0.001). Maximum peak-to-peak displacements for uncorrected and corrected acquisitions without induced motion were found to have statistically significant differences (*p*-value < 0.001).

#### 3.4.1 | Secondary Analysis of Peripheral Motion

Analysis of the baseline data used in this study showed that the peripheral tissue exhibited non-negligible displacement magnitudes relative to the interior when motion correction was not applied (Figure S1A). After motion correction was applied, interior displacement magnitudes exhibited variable reductions across participants, whereas peripheral displacement magnitudes experienced significant reductions in all participants (Figure S1B). In some participants, motion in the periphery did show a pattern of motion synchronized with the cardiac cycle (Figure S2, Participant A). This pattern was typically highest in magnitude in the S/I direction. However, in the majority of participants, cardiac-synchronous fluctuations in the periphery were not observed (Figure S2, Participant B).

## 4 | Discussion

Test–retest DENSE scans were acquired on 10 participants at three different slice positions in the brain, with and without induced motion. Scans were processed using three post-processing pipelines, to assess the effect of motion on cranial DENSE scans and the effectiveness of current motion correction methods. The main post-processing pipeline included standard post-processing steps used in prior work, and the two additional pipelines included an added subtraction step informed by the outer ring of brain tissue. Results processed without additional motion correction demonstrated qualitative differences in displacement maps and were found to have statistically significant differences in peak-to-peak displacement measures (*p*-value < 0.001), demonstrating bulk motion corrupts DENSE results and leads to an overestimate of displacement. Improved correspondence in both qualitative and quantitative measures between scans acquired with and without induced motion was observed with the addition of a subtraction of a linear polynomial fit of the phase in the outer ring of brain tissue. Performing the subtraction using a complex polynomial fit was more robust to phase wrap, demonstrated the greatest improvement in correspondence between scans acquired with and without induced motion, and showed the highest repeatability. Motion correction pipelines reduced the measured displacement, even in the absence of induced motion, suggesting either artificial suppression of cardiac-induced brain motion or removal of motion from non-cardiac sources in cooperative participants.

As DENSE is susceptible to sub-millimeter amounts of bulk patient motion, motion reduction and correction is necessary. Many prior studies have focused on technical development, utilizing cohorts of younger healthy and cooperative participants, minimizing the potential for large amounts of bulk patient motion observed in clinical populations. Options for reducing bulk patient motion during brain imaging include head immobilization devices or additional head supports (i.e., padding), but these do not guarantee a lack of bulk motion. Additional head supports in the head coil were used in this study, in scans with and without induced motion, and yet motion correction methods reduced measured displacement in both, suggesting padding does not sufficiently prevent both large and small amounts of bulk motion. This study suggests that motion correction, not just preemptive reduction, may be a requisite step in all post-processing of DENSE brain scans, to remove both large and small amounts of bulk motion not adequately prevented through other means. Without motion correction methods, DENSE imaging in the brain is vulnerable to incidental bulk motion, greatly limiting its implementation clinically.

Various MR-based methods exist to measure cardiac-induced brain tissue motion, including DENSE [81–90], PC-MRI [31, 32, 91–94], and quantitative-amplified MRI (q-aMRI) [95–100]. DENSE can measure displacement with high spatial and temporal resolution, be tuned to be sensitive to cardiac-induced brain motion and directly provide quantitative measures. Phase-Contrast MRI (PC-MRI) uses bipolar gradients to encode tissue velocity information into the phase of the MR image and, similar to DENSE, requires encoding in multiple directions leading to longer scan times. Displacements cannot be directly measured with PC-MRI and are instead estimated by integrating the measured velocities over time, which can introduce additional errors, with the potential effects of bulk head motion largely uninvestigated. The results in this study suggest that due to the high level of motion sensitivity needed for cardiac induced brain motion, each individual excitation requires correction. This precludes the use of traditional CINE averaging over multiple heart beats. Quantitative-amplified MRI visualizes pulsatile brain tissue motion over the cardiac cycle by amplifying subtle temporal intensity changes in cardiac-gated cine MRI data, and boasts pronounced tissue contrast, short scan times and high spatial and temporal resolution. However, it uses an optical-flow algorithm [100] to estimate sub-voxel motion on the order of 0.01 pixel size, requiring high resolution images (1.2–1.8 mm isotropic) in order to accurately measure subtle brain tissue motion. Again, the acquisition of these images will require averaging over multiple heart beats and there remains potential for bulk head motion to lead to errors in the displacement field. Further, the optical-flow algorithm requires tuning of various parameters, such as image resolution and dimension, Gaussian filter (*σ* value), and temporal frequency band, all of which significantly impact the amplitude of the extracted brain motion field.

The motion correction method used in this study relied solely on the acquired DENSE data to estimate and correct for bulk head motion. The proposed outer ring subtraction method is similar to an approach taken in Poncelet et al. [92]. Poncelet et al. measured velocities encoded in the phase data, isolated the rigid motion of the skull, and subtracted it from the brain parenchyma velocity maps, thus “remov[ing] the instantaneous contribution of rigid head motion on a frame-by-frame basis”. While the approaches differ in which area is segmented out for the polynomial fit (skull for Poncelet et al., outer ring of brain tissue for this work) and encoded information (velocities for Poncelet et al., displacements for this work), both involve the segmentation of a region presumed to remain stationary under ideal circumstances (i.e., no patient bulk motion or physiological motion expected in that region), which is then fit to a polynomial and subtracted from the original phase of the image. In this study, segmentation of the “stationary” signal in the skull was not feasible due to inadequate signal-to-noise ratio (SNR) in that region.

Other methods for tracking and estimating head motion, for the purposes of retrospective motion correction, include fat navigators [118–123] and optical tracking methods [123–132]. To track the motion between DENSE images taken over a cardiac cycle using fat navigators, navigators would have to be interspersed between the DENSE echoes in the readout train. Inclusion of fat navigators would increase the minimum repetition time (TR) between DENSE echoes, decreasing the number of DENSE echoes that can be acquired over a single cardiac cycle. Additionally, alternating between fat and water selective excitation pulses would disrupt the natural steady-state equilibrium of the system, leading to potential SNR reductions when low SNR is already a concern in DENSE sequences. Optical tracking methods would provide a direct measurement of head motion without impacting the timing or signal of the DENSE sequence, but they require additional hardware, initial setup and calibration, and additional processing time, increasing the barrier for clinical implementation. While the accuracy of marker-based optical tracking may be a few tens of microns in bench-top experiments, it relies heavily on a perfectly rigid fixation of the physical markers to the head.

While the method proposed in this study benefits from not requiring additional echoes or hardware, it does come with limitations. It is based on the assumption that the outer ring of brain tissue is not subject to cardiac-induced motion, so any motion within that region would be attributable to either bulk motion or other non-cardiac physiological sources (e.g., respiration). Prior work supports that the largest cardiac-induced displacements occur in deeper brain regions rather than along the periphery [83–86, 115]. In baseline data without motion correction, we observed non-negligible displacements in the outer ring relative to those in the interior. In some participants, we observed synchronization of the peripheral motion with the cardiac cycle. Thus, the outer ring polynomial correction removes motion that is cardiac-related. However, it remains unclear whether this motion reflects movement of the brain within the skull or represents a bulk motion effect arising from force propagation during the cardiac cycle. Prior work in heartbeat detection using video imaging [133] and head cardioballistograms [134] has shown that cardiac motion can be measured from peripheral motion outside of the skull. This, along with the fact that the motion is well corrected for using linear polynomials, suggests that outer ring motion likely includes cardiac-induced bulk motion, although further work is needed to disentangle this from motion occurring within the skull, due to cardiac or other non-cardiac physiological sources (e.g., respiration). This study solely used photo-plethysmography (PPG) gating and did not record additional physiological signals (e.g., respiratory or ECG). The absence of respiratory monitoring limited our ability to determine whether any of these peripheral fluctuations might reflect respiratory-related motion. Recent work directly imaging respiration-induced brain motion using DENSE has shown that respiration induces an inferior translation of the complete head accompanied by a slight anterior rotation [89]. Such components of motion would likely be removed by our motion correction method. Since we are interested in imaging and measuring cardiac-induced motion of the brain tissue, removal of the bulk and non-cardiac physiological motion is ideal for our purposes. Further research is needed to determine whether the reduction in displacement measures observed without purposefully induced bulk motion after correction is artificial suppression of brain tissue motion due to the polynomial being fit to a subset of brain tissue, and/or merely a consequence of correcting incidental bulk motion and non-cardiac physiological brain motion during standard scans. Previous work using MR-based methods have estimated displacements on the order of 0.03–0.09 mm in the regions of the brain imaged in this study [82–84, 91, 112, 135]. Peak-to-peak displacements measured in this study, after motion correction methods were applied (Complex Fit Pipeline), were 0.04 ± 0.01 mm (mean) to 0.12 ± 0.04 mm (maximum), which fall within the range of previous estimates, despite the observed reduction in displacement measures in the absence of purposefully induced motion. Whether these prior reported displacements would be similarly affected (i.e., reduced) by the motion correction method presented here remains unclear. Future work would benefit from recording additional physiological signals (e.g., respiratory or ECG data) to better characterize contributions from non-cardiac motion sources.

Another limitation of the study was the number, positioning and thickness of slices. The number of slices was kept limited to keep total scan time relatively short (~30–35 min) and slice positions were spaced further apart to test the reproducibility of the motion correction method’s effectiveness within multiple regions of the brain. Since slice positions were not directly adjacent to each other, slice-to-slice patterns in motion couldn’t be compared, which may offer additional insight into the sources of motion within the outer ring of brain tissue prior to correction. The thickness of the slices was chosen to be 10 mm for SNR purposes, making the results susceptible to partial volume effects.

Participant heartrates on average tended to increase between acquisitions taken without motion (baseline) and acquisitions taken with motion, which caused misalignments in some scans between the actual TR (calculated at time of prescription) and the ideal TR (calculated retrospectively using PPG data) (Figure S3A). Heartrate variability, assessed using the RMSSD, may be a confounding factor. One participant, exhibiting consistently higher heartrate variability than the others, showed a lower overall correlation (*R*^2^) between induced motion and baseline scans after correction, in contrast to the other participants, who had relatively stable *R*^2^ values despite variations in heartrate variability (Figure S3B).

Additional limitations of the study were the population it was performed on and the method of induced motion. This study was performed on a cohort of young and healthy participants, for the purposes of validating the motion correction method, before implementation in broader populations can be considered. The induced motion utilized (continuous alternating plantar flexion and dorsiflexion of the feet) was chosen because it is controlled, repeatable, and both large enough to produce motion effects in the data while remaining small enough to be realistic for patient cohorts. The pattern of induced motion will not exactly replicate motion observed in clinical practice. Future work is needed to validate the effectiveness of the proposed motion correction method within patient cohorts before it can be used in clinical applications.

## 5 | Conclusions

DENSE scans acquired during purposefully induced motion showed marked differences when compared with those without induced motion, including residual phase wrap artifacts within generated displacement maps, reinforcing the need for adequate motion correction methods. Implementing the subtraction of a linear polynomial fit to the phase of the voxels in the outer ring of brain tissue from the overall phase image substantially improved the correspondence between, and repeatability of, DENSE measures acquired with and without purposefully induced bulk motion. The greatest improvement was observed when the subtraction was performed in the complex domain, which accounted for and removed uncorrected phase wrap caused by purposefully induced bulk motion. Future work is needed to validate the effectiveness of the proposed motion correction method within patient cohorts before it can be used in clinical applications.

## Supplementary Material

Supporting Information

Additional supporting information can be found online in the Supporting Information section. Table S1: Table summarizing the quantitative results from each of the three pipelines compared in study, particularly the Linear Fit Pipeline results, which were not included in the main text. Figure S1: Boxplots displaying the peak-to-peak displacement magnitudes in baseline scans across slices within each participant for the outer ring (blue) and the interior brain tissue (red). Before motion correction (A), the displacement in the outer ring is on the order of the motion within the brain tissue. This is despite the participants efforts to remain still. After motion correction (B), the displacement in the outer ring is reduced to near noise levels while the motion within the brain remains higher. Figure S2: Displacement curves from outer ring of brain tissue in two participants. Four averages from the scan were reconstructed separately and plotted as separated curves for comparison. Each curve is composed of a transparent region representing the range of displacements at each time point, and a bold line representing the mean displacement value at each time point. In participant A (participant 2 in Figure S1), there is clear cardiac driven motion that is observed in the superior/inferior direction that is consistent among the repeated averaged. In participant B (participant 10 in Figure S1) and in other directions, the displacement lower and less correlated among the repeated scans. This suggests the outer ring does contain motion that is cardiac in nature. However, this analysis is not able to determine if this motion is cardiac induced bulk motion of the entire head or if this is motion of brain tissue separate from the skull motion. Figure S3: (A) Ideal TR (calculated retrospectively using the PPG data from the scan) plotted against the Actual TR (calculated at time of prescription). (B) R2 changes with respect to the heartrate variability (RMSSD). Each participant is represented by a distinct color; all data points sharing the same color correspond to the same individual. In part B, RMSSD values were computed for each scan and averaged across the paired acquisitions used to calculate the corresponding R2 value.

## Data Availability

The data that support the findings of this study are available from the corresponding author upon reasonable request.
