# Supplementary material for: Effects and Correction of Patient Bulk Motion in Cranial DENSE MRI
Source: Magn Reson Med. 2026 Feb 1;95(6):3145–60. doi: 10.1002/mrm.70270 (PMC13023016; doi:10.1002/mrm.70270)
Supplement: Supplementary file 1 — Table S1: Table summarizing the quantitative results from each of the three pipelines compared in study, particularly the Linear Fit Pipeline results, which were not included in the main text. Figure S1: Boxplots displaying the peak‐to‐peak displacement magnitudes in baseline scans across slices within each participant for the outer ring (blue) and the interior brain tissue (red). Before motion correction (A), the displacement in the outer ring is on the order of the motion within the brain tissue. This is despite the participants efforts to remain still. After motion correction (B), the displacement in the outer ring is reduced to near noise levels while the motion within the brain remains higher. Figure S2: Displacement curves from outer ring of brain tissue in two participants. Four averages from the scan were reconstructed separately and plotted as separated curves for comparison. Each curve is composed of a transparent region representing the range of displacements at each time point, and a bold line representing the mean displacement value at each time point. In participant A (participant 2 in Figure S1), there is clear cardiac driven motion that is observed in the superior/inferior direction that is consistent among the repeated averaged. In participant B (participant 10 in Figure S1) and in other directions, the displacement lower and less correlated among the repeated scans. This suggests the outer ring does contain motion that is cardiac in nature. However, this analysis is not able to determine if this motion is cardiac induced bulk motion of the entire head or if this is motion of brain tissue separate from the skull motion. Figure S3: (A) Ideal TR (calculated retrospectively using the PPG data from the scan) plotted against the Actual TR (calculated at time of prescription). (B) R2 changes with respect to the heartrate variability (RMSSD). Each participant is represented by a distinct color; all data points sharing the same color correspond to the same [file MRM-95-3145-s001.docx]

***Supplemental Table S1:*** *Table summarizing the quantitative results from each of the three pipelines compared in study, particularly the Linear Fit Pipeline results, which were not included in the main text.*

|  | Standard Pipeline | Linear Fit Pipeline | Complex Fit Pipeline | Associated Figure |
| --- | --- | --- | --- | --- |
| R^2^ values between induced motion and baseline pairs | 0.45 ± 0.29 | 0.77 ± 0.21 | 0.96 ± 0.05 | Figure 4A |
| RMSD values between induced motion and baseline pairs | 0.089 ± 0.005 mm | 0.0203 ± 0.0003 | 0.00788 ± 0.00004 | Figure 4B |
| Mean Spearman’s Rank Correlation coefficients between test-retest datasets | 0.56 ±.01 | 0.83 ± 0.07 | 0.977 ± 0.004 | Figure 6 |
| Spearman’s Rank Correlation Coefficients between test-retest datasets in data with induced motion | 0.46 ± 0.38 | 0.76 ± 0.19 | 0.98 ± 0.01 | Figure 6 |
| Spearman’s Rank Correlation Coefficients between test-retest datasets in data without induced motion | 0.76 ± 0.35 | 0.97 ± 0.05 | 0.98 ± 0.02 | Figure 6 |

***Supplemental Figure S1:*** *Boxplots displaying the peak-to-peak displacement magnitudes in baseline scans across slices within each participant for the outer ring (blue) and the interior brain tissue (red). Before motion correction (A), the displacement in the outer ring is on the order of the motion within the brain tissue. This is despite the participants efforts to remain still. After motion correction (B), the displacement in the outer ring is reduced to near noise levels while the motion within the brain remains higher.*

*
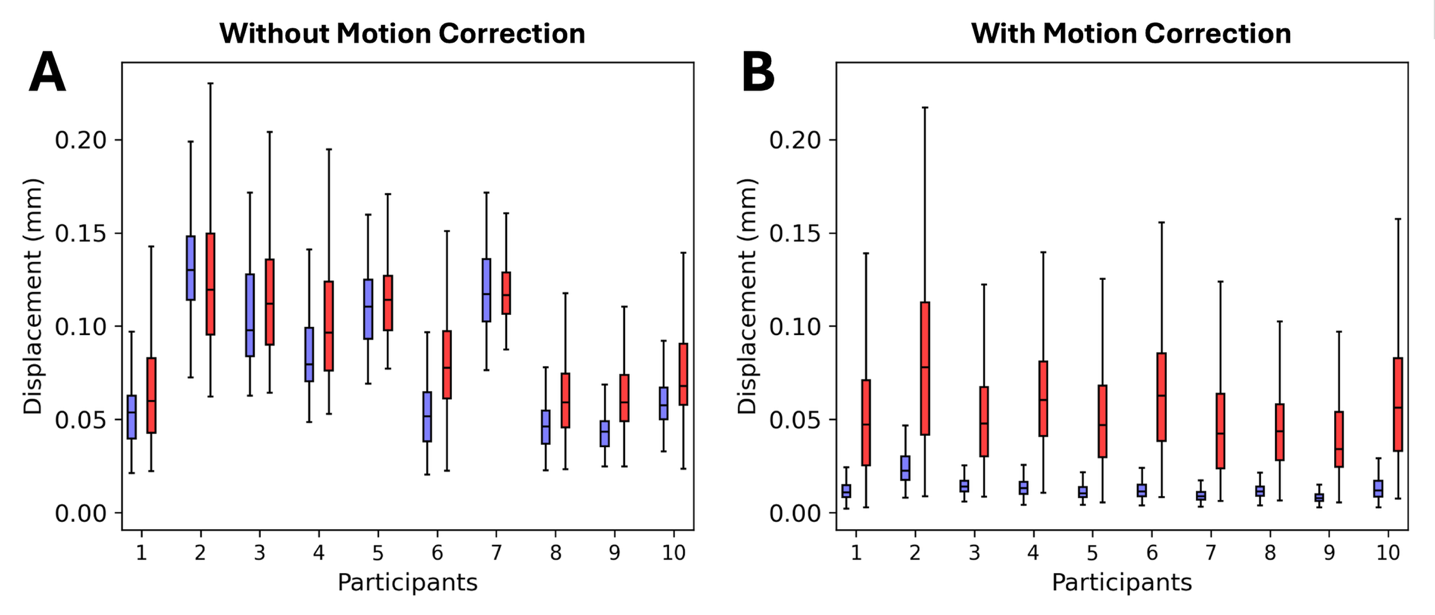
*

***Supplemental Figure S2:*** *Displacement curves from outer ring of brain tissue in two participants. Four averages from the scan were reconstructed separately and plotted as separated curves for comparison. Each curve is composed of a transparent region representing the range of displacements at each time point, and a bold line representing the mean displacement value at each time point. In participant A (participant 2 in Supplementary Figure S2), there is clear cardiac driven motion that is observed in the superior/inferior direction that is consistent among the repeated averaged. In participant B (participant 10 in Supplementary Figure S2) and in other directions, the displacement lower and less correlated among the repeated scans. This suggests the outer ring does contain motion that is cardiac in nature. However, this analysis is not able to determine if this motion is cardiac induced bulk motion of the entire head or if this is motion of brain tissue separate from the skull motion.*

***
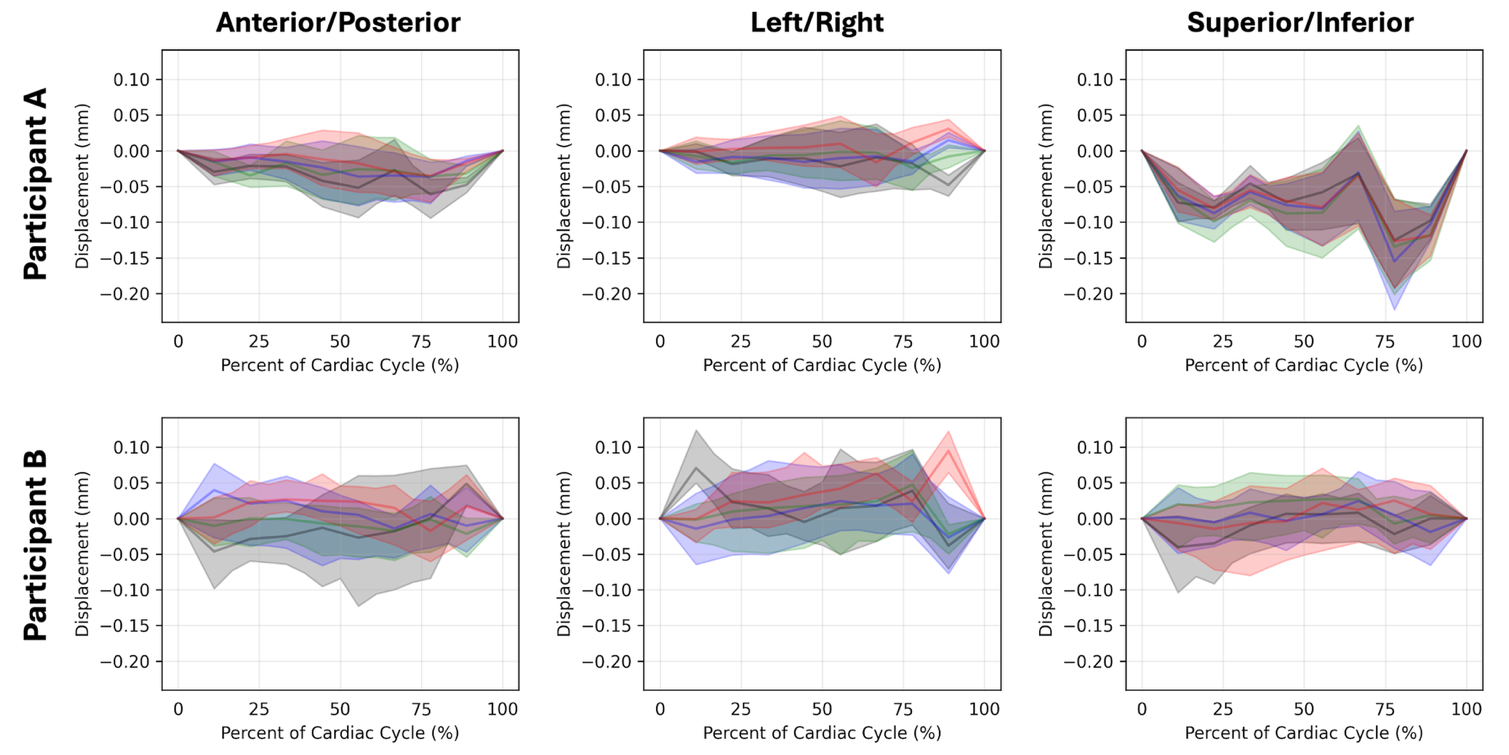
***

***Supplemental Figure S3:*** *A. Ideal TR (calculated retrospectively using the PPG data from the scan) plotted against the Actual TR (calculated at time of prescription). B. R^2^ changes with respect to the heartrate variability (RMSSD). Each participant is represented by a distinct color; all data points sharing the same color correspond to the same individual. In part B, RMSSD values were computed for each scan and averaged across the paired acquisitions used to calculate the corresponding R² value.*

*
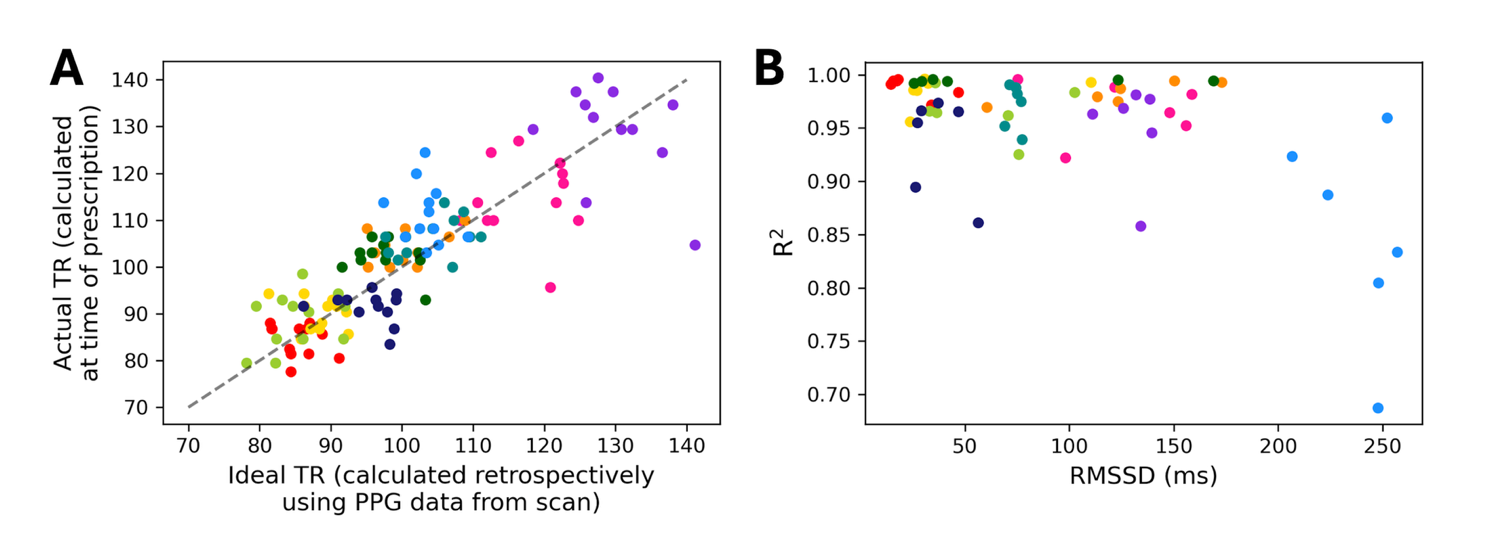
*
